# Role of Partial Splenectomy in Hematologic Childhood Disorders

**DOI:** 10.3390/pathogens10111436

**Published:** 2021-11-05

**Authors:** Giorgio Attina’, Silvia Triarico, Alberto Romano, Palma Maurizi, Stefano Mastrangelo, Antonio Ruggiero

**Affiliations:** Pediatric Oncology Unit, Department of Woman and Child Health and Public Health, Fondazione Policlinico Universitario A. Gemelli IRCCS, Università Cattolica del Sacro Cuore, 00168 Rome, Italy; giorgio.attina@policlinicogemelli.it (G.A.); silvia.triarico@guest.policlinicogemelli.it (S.T.); albero.romano90.ar@gmail.com (A.R.); palma.maurizi@unicatt.it (P.M.); stefano.mastrangelo@unicatt.it (S.M.)

**Keywords:** partial splenectomy, children, prophylaxis, sepsis, hematologic disorders, vaccinations

## Abstract

The spleen is a secondary lymphoid organ that belongs to the reticular-endothelial system, directly connected to blood circulation. The spleen is greatly involved in the immune response, especially against capsulated bacteria. Splenectomy plays a fundamental role in the treatment of numerous pediatric hematologic disorders. Taking into account all the possible complications (especially infections) linked to this procedure, alternatives to total splenectomy have been sought. Partial splenectomy has been proposed as a treatment that allows the reduction of infectious risk. This approach has proven safe and feasible in most patients, but multicentric and prospective studies are necessary to more accurately define the indications for performing partial splenectomy. However, vaccinations and antibiotic prophylaxis remain fundamental for preventing serious infections, even in the case of partial splenectomy. We review anatomical and functional properties of the spleen, with a focus on medical or surgical indications to splenectomy, aiming to give practical educational information to patients and their families after splenectomy. Furthermore, we discuss the feasibility of partial splenectomy in children with hematologic diseases who require splenectomy.

## 1. Introduction

Currently splenectomy is still fundamental in the treatment of numerous pediatric hematologic and immunological disorders. The main indications for splenectomy are represented by congenital hemolytic anemia (hereditary spherocytosis, elliptocitosis, pyruvate kinase deficiency, drepanocytosis), some forms of thalassemia, idiopathic thrombocytopenic purpura not responsive to medical treatment, and some forms of accumulation disease associated with hypersplenism (Gaucher’s disease, Niemann-Pick’s disease) [1]. There are also surgical indications, such as the presence of splenic tumors or cysts, abscesses, trauma with partial and or total rupture. In all these situations splenectomy plays a potentially resolving therapeutic option. However, it may be burdened by very important infectious and vascular complications that often limit its indication or therapeutic success [2,3].

In our work, we review anatomical and functional properties of the spleen, performing a focus on medical or surgical indications to splenectomy, with the aim to give a practical educational guide to patients and their families after splenectomy. Furthermore, we discuss the opportunity of partial splenectomy that in recent years has been considered to be a feasible alternative to radical splenectomy in order to obtain a clinical resolution, reducing or eliminating the infectious risks related to the absence of the spleen, which plays an important role in the immune response, especially against capsulated bacteria.

## 2. Anatomy and Functions of the Spleen

The spleen is a secondary lymphoid organ that is part of the reticular-endothelial system. Its peculiarity is given by its faster and more immediate action, because of it is connected to the blood circulation directly and not through the lymphatic vessels like the other lymphoid organs [4]. The spleen is located in the abdominal cavity, at the level of the 10th rib in the left hypochondrium, between the gastric bottom and the diaphragm. It has a diaphragmatic face, smooth and convex, in contact with the abdominal face of the diaphragm, and a visceral face in contact with the back wall of the stomach, with the upper pole of the left kidney and sometimes with the left adrenal, with the left colic flexure and the tail of the pancreas. The spleen is almost surrounded by the peritoneum that adheres tenaciously to its capsule. The vascularization of the spleen is ensured by the splenic artery, a branch of the celiac trunk, which enters the organ at the level of the hilum and then divides into central arteries and arterioles. The size and weight of the spleen vary according to age and may vary depending on different situations in the same individual. On average, in the adult the spleen measures 10–12 cm of length, 7 cm of width, 3–4 cm of thickness and weighs on average 150 g. Often, in proximity to the spleen or more rarely, at a distance throughout the abdomen, small encapsulated nodules of splenic tissue, called accessory spleens, can be found. They may be isolated or in continuity with the spleen through thin splenic tissue bridges. This finding is present in 10–15% of subjects. From the embryonic point of view, it develops starting from the 6th week of intrauterine life with the formation of multiple nodules that originate from the celomatic epithelium and the mesenchyme of the dorsal mesogastrium. These nodules then merge giving the initial lobular appearance of the spleen. The vascular network is well-developed toward the 8th–9th week, while between the 11th and the 12th week the differentiation of blood cells, macrophages and arteries, veins, capillaries and sinusoids are completed and the spleen is thus ready to produce blood cells.

**Figure 1:**
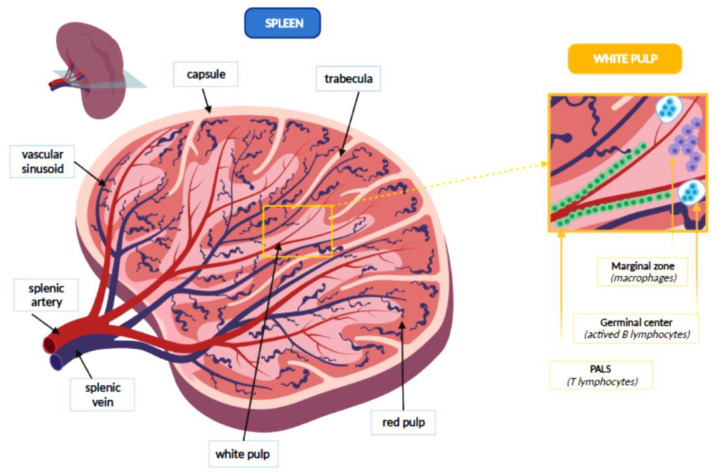
Anatomical features of the spleen.

The spleen is covered by the capsule, a fibrous lining from which some trabeculae branch to form a net that constitutes the splenic stroma. Inside this network there are the red and the white flesh, at the margins of which the fibrous reticulum thickens, forming the marginal zone. The white pulp represents about the 25% of the splenic tissue and consists entirely of lymphoid tissue, which may be divided into three sections: periarteriolar lymphoid sheaths (PALS), germinal centers and marginal zone. Splenic white pulp is organized around central arteries in the form of PALS, which are populated mainly by T lymphocytes. Germinal centers contain lymph follicles with actived B lymphocytes that produce antibodies, following an antigenic stimulus. Finally, the marginal zone develops between the white pulp and red pulp. It contains antigen-presenting cells (APCs), such as macrophages. Splenic arterioles then cross the marginal zone where they thicken macrophages and fibroblasts and finally reach the red pulp, which represents 75% of the spleen. In the red pulp, they form venous sinusoids which are surrounded by a tissue rich in macrophages and are internally covered by endothelial cells. Outside of these venous sinuses, the red flesh is structured in the cords of the spleen, a network of reticular fibers, fibroblasts and macrophages with high phagocytic activity. Figure 1 represents the main anatomical features of the spleen [5].

Numerous body functions are carried out in the spleen, such as phagocytosis, hematopoiesis, deposition and immune response. Splenic macrophages show a high phagocytic capacity, due to the presence of lysosomes rich in enzymes very active in hydrolysis and destruction of tissue debris, corpuscular substances and opsonized infectious agents. In addition to these cells there are numerous mononucleate dendritic phagocytes which are only moderately phagocytic. They perform the function of antigen-presenting cells, by activating the response of T and B lymphocytes, which are present in the white flesh and then migrate in the splenic cords and the bloodstream [6]. The macrophages of the spleen also perform haematocatheretic function against old, damaged or altered blood cells. As the red blood cells are destroyed, bilirubin is channeled through the blood flow to the liver for the excretion. The sphere is widely reused at the bone marrow level, while iron is recycled at the bone marrow level. Splenic phagocytes may remove some intracytoplasmic inclusions of red blood cells (Howell-Jolly bodies, erythrocyte pits), without destroying entirely the red blood cell. The role of hematopoiesis is played during the four months of intrauterine life and usually ends after birth, although in some particular situations, the stem cells that persist in the red pulp even after birth may resume a hematopoietic function. In addition, the spleen has a lymphopoietic function, which helps to form a circulating reserve of T and B lymphocytes and mononucleate phagocytes. A further role of the spleen is the blood deposit, thanks to the rich vascularization of this organ that may filter 150 mL of blood per minute. In some cases, such blood its blood cells can be poured into circulation in response to particular situations (anemia, bleeding), following adrenergic stimulation that causes the contraction of the spleen. Thus, the spleen is a fundamental organ in both innate and adaptive immune response, but it also determines the clinical manifestations of certain congenital and acquired hematologic disorders (such as anemia, thrombocytopenia) that may require its removal [5].

## 3. Splenectomy

Splenectomy is a surgical procedure that is performed on about 6.4–7.1 people per 100,000 each year. About 25% of splenectomy interventions are performed as a result of abdominal trauma that causes rupture of the spleen resulting in intraabdominal bleeding and risk of hemodynamic shock. Another 25% of operations are performed for the treatment of hematologic disorders. Less frequent causes of splenectomy are consequent to intrinsic injuries of the spleen, tumors, abdominal surgery, accumulation or vascular disease [7]. A study conducted by the “Agency for Healthcare research and quality healthcare cost and utilization” showed a slight decrease in the number of splenectomy interventions performed each year from 1993 to 2014 in the USA, probably linked to the development of alternative therapies for hematologic diseases, such as rituximab, thrombopoietin receptor agonists, and immuno-acting agents. Despite this, splenectomy still remains an important therapeutic option in a lot of medical and surgical disorders [8], as described in Table 1.

In splenectomized patients, a significant problem is represented by overwhelming post-splenectomy infections (OPSI) that are characterized by a sudden and rapid general deterioration that occurs after about 24 h from the onset of the first infectious sign, such as flu, up to the framework of fulminating sepsis with CID and septic shock that can quickly lead to death [9,10]. The incidence of sepsis in splenectomized children is currently between 1.8–4%, with a mortality that can reach about the 50% of cases. This incidence is at a maximum in the first three years after the splenectomy and then is progressively reduced, persisting throughout the life span and being able to occur even after 50 years. In the first five years of life, a risk between 60 and 100 times greater than non-splenectomized subjects is estimated [11].

The age of the child (maximum under 2 years and however high up to 5 years), the time elapsed from the splenectomy (greater risk in the first 2–3 years that then decreases without ever zeroing and resurfacing after 60 years), the basic disease (drepanocytosis, thalassemia and some neoplastic pathologies and in traumas) are known as risk factors for OPSI [12,13,14]. 

Splenectomy should therefore be avoided up to five years of life, when possible. OPSI are caused mainly by capsulated bacteria (Streptococcus pneumoniae in 50% of cases, to a lesser extent Haemophilus Influenzae type b, and Neisseria Meningitides) [15,16]. In addition, splenectomized children have a higher risk of developing severe forms of Malaria, Bordetella, Babesiosis and secondary dog bite infections (such as Capnocytophaga canimorsum) [17,18].

The OPSI are burdened by a high mortality risk, even if treated appropriately, therefore a prevention program is crucial in splenectomized subjects, based on three pillars: family education, vaccination, and antibiotic prophylaxis [19]. 

Parents should be informed about the infectious risk and should be made aware of the necessity of always starting quickly an antibiotic therapy at the first signs of infection, and going to the hospital for a rapid medical evaluation at the onset of symptoms. It should also be recommended to avoid areas where malaria is endemic, paying particular attention to dog bites. Several studies showed that splenectomized individuals and their family members have very little awareness about the risks related to their condition. It has also been reported that patients with more comprehensive and adequate information, have a lower risk of presenting OPSI [20]. Table 2 shows the most important issues for the family and patients’ education.

Vaccination is the most effective prevention tool for splenectomized patients. Children should be vaccinated two weeks before the splenectomy, when the surgery is planned electively, as in the case of hematologic disorders. In those cases when the splenectomy is performed in an emergency, vaccination should be carried out from 14 days after surgery, although some data suggest that they can be effective even in the immediate peri-operative time [21].

There is a fair variability in the recommendations regarding the type of vaccination and the precise timing to follow, also related to the history of vaccination and the age at the time of splenectomy of the subject. Most of the guidelines indicate vaccinations against the main capsulated germs, such as Pneumococcus, Meningococcus and Hemophilus Influenzae type B, and annual anti-flu vaccination [22].

Conjugated vaccines that seem to provide a better immunological response in splenectomized subjects are generally indicated, since these vaccines stimulate a response mediated by thymic T cells that is not modified by the splenectomy [23].

Concerning anti-pneumococcus vaccination, it is recommended to perform a dose of conjugate vaccine (PCV13) followed by a boost performed with polysaccharide vaccine (PPSV23). The vaccination against meningococcus should include the administration of both the conjugated vaccine quadrivalent (Men A-C-W-Y) and the most recent conjugated vaccine against serotype b (Men B). It has not yet been clarified the indication to perform a recall after the first vaccination, although several recommendations suggest a recall every 3–5 years. For vaccination against Hemophilus Influenzae, a single dose is indicated without any recall in previously vaccinated subjects. Although influenza virus infection does not pose increased risks in splenectomized subjects, annual vaccination is indicated to reduce the risk of any bacterial infection, performed with inactivated vaccines and not with attenuated vaccines [24].

In splenectomized patients, no increased risk of developing severe Sars-Cov2 infections was reported. In a study on asplenic patients in England, the number of deaths from coronavirus disease was not statistically different from the general population [25,26].

After splenectomy, for children who have performed an adequate administration of vaccines, continuous antibiotic prophylaxis is recommended, which has been shown to reduce the risk of severe infection by 50% and death by about 90% [27].

Adherence to vaccination recommendations has proved very variable and unsatisfactory in most splenectomized subjects [28]. Moreover, a vaccine failure or an infection caused by a pathogen other than those provided by the vaccination coverage (Pseudomonas Aeruginosa, Escherichia Coli) may occur even in those who appropriately follow the proposed vaccination schedule. Orally administered penicillin is the indicated antibiotic. Although for the recent outbreaks of resistant bacteria, amoxicillin-clavulanic acid and trimethoprim-sulfamethoxazole or, in allergic subjects, erythromycin, are now recommended. There is less consensus on the duration of the above-mentioned prophylaxis. It is common to advise for the administration of antibiotic prophylaxis in the first 2–3 years after the intervention, and often up to 5 years of age: however, some authors and some centers recommend administration at least until adulthood (16–18 years) and in some studies even for life. There are issues regarding the stop of prophylaxis by the patient and the risk of the onset of antibiotic resistance. Life-long prophylaxis is also suggested for subjects who have presented an episode of sepsis or have particular risk factors. Splenectomy has been associated with a higher risk of thromboembolic events, such as portal vein thrombosis, deep venous thrombosis and pulmonary embolism and in some studies the appearance of pulmonary hypertension. Another problem that may arise after splenectomy is thrombocytosis, which can be transient or prolonged. In the cases of prolonged thrombocytosis with platelet values higher than 1.000.000/mmc the antithrombotic prophylaxis with acetylsalicylic acid is indicated [29].

Splenectomy may generate a prothrombotic state, with consequent increased risk of venous thromboembolic disease, such as thromboembolic pulmonary hypertension. Thus, splenectomy is recognized as a potential risk factor for chronic thromboembolic pulmonary hypertension (CTEPH) [17]. Recently, *Zhang* et al. performed a systematic review and meta-analysis to explore the association between splenectomy and CTEPH, finding a prevalence of splenectomy in CTEPH of about 4% [30]. 

From a surgical point of view, the laparoscopic approach is preferable as it reduces intraoperative complications, post-operative pain and hospitalization time. However, the laparotomic approach is however still widely performed and accepted especially in the cases of important splenomegaly. No differences were found in the results of the hematological response, nor in the infectious and thromboembolic complications. In recent years, to reduce the main infectious risks related to total splenectomy, the indication to partial splenectomy has become more important, even though it was performed for the first time in the 60 s. Currently, the improvement of surgical techniques has allowed to extend the laparoscopic approach to this procedure [31]. Partial splenectomy consists of removing 70–80% of the spleen, as it has been shown in experimental models that the remaining 25–30% of the initial splenic tissue may ensure its immune action. The benefits of a partial splenectomy could be greater in patients with a high risk of infection, since the residual splenic tissue could ensure the desired hematologic effect, maintaining a good immunological function and reducing the risk of severe sepsis in children. Despite these assumptions, current data are limited, not allowing to clearly define the risk/benefit ratio of this procedure [32].

## 4. Discussion

The main indications for splenectomy can be medical or surgical. In some cases, as in hereditary spherocytosis, hereditary ellipsocitosis, and some lesions of the spleen, it represents the only therapeutic option. In other diseases, it must be evaluated according to the clinical characteristics of the patient. Splenectomy is considered possible and safe in most children for whom it is indicated, but regarding partial splenectomy, although most of the existing studies are multicentric or performed by single centers, the number of patients is small and it does not allow having clear indications for it. The main advantage of partial splenectomy is the preservation of residual splenic tissue that can maintain the immune function of the spleen, reducing the risk of fulminating sepsis. It is very difficult to extrapolate from the literature to what extent this risk is reduced by subjecting the child to a partial splenectomy, compared to the total one, because there are no trials large enough or with an extended follow-up. In addition, most of these studies were performed before the diffusion of the antistreptococcal vaccine 13-valent and meningitis vaccines, so the actual incidence of post-splenectomy sepsis is difficult to clarify [33].

In some works, the production of total Immunoglobulin G (IgG) and M (IgM) was evaluated and it seems to be stable after the intervention of partial splenectomy. The spleen remains the main antibody production site, so this data suggests the importance of the action of the residual spleen [34].

Sheikha AK et al. collected data from 62 children affected by hereditary spherocytosis, who underwent partial splenectomy. They found a significant improvement in hematologic profiles and symptoms in all patients, briefer in those with a good splenic post-operative regeneration. Moreover, none of them developed sepsis, demonstrating that partial splenectomy may be effective in reducing the OPSI events, becoming an effective alternative in children with hereditary spherocytosis [35].

Regarding the hematologic response to partial splenectomy in patients with spherocytosis, there was a satisfactory improvement in the hemoglobin values, even if lower than that found in patients subjected to total splenectomy (an increase of the hemoglobin of 2.4 g/dl against 4.1 g/dl). Moreover, there was a reduction of the values of reticulocytes and bilirubin overlapped to those obtained from patients who underwent total splenectomy. In drepanocytosis, there was no increase in hemoglobin values, but an improvement in the clinical picture with fewer symptoms related to hypersplenism and splenic size. In subjects with immune thrombocytopenia, there are still no clear data on the short-term and long-term outcomes of partial splenectomy. In such a pathology, the use of partial splenectomy could be limited, considering the hemorrhagic risks related to thrombocytopenia, even if there was no evidence of a different bleeding rate between the partial and total splenectomy. It was necessary to convert the surgery from laparoscopic to laparotomic more often in the partial than in the total splenectomy (30% vs. 1–3%). Apart from this, perioperative complications were minimal and not significantly different from total splenectomy [36,37,38,39,40,41].

One aspect to consider is the rate of conversion from partial splenectomy to total splenectomy during surgery, for the following days and even the following months [42]. In the literature, there are no clear data: in multiple case studies a frequency of 0% to 42% is reported, although more frequently, 5–10% is reported. Romboli et al. performed a literature review of about 457 cases of laparoscopic partial splenectomy performed for hematologic disease (hereditary spherocytosis and drepanocytosis), splenic focal masses and trauma. They found no mortality, low morbidity, and low conversion rates to laparotomy partial splenectomy. Furthermore, they reported 17 cases out of 147 (3.7%) that required the conversion of splenectomy from partial to total; five of them for surgical complications during the intervention and 12 for therapeutic failure after the surgery [43].

Because of the risk of conversion to total splenectomy and in the absence of clear data about the contribution of the residual splenic tissue to the reduction of the risk of sepsis, the indication to pre-intervention vaccination and post-antibiotic prophylaxis should be assessed in more extensive studies. Furthermore, cholecystectomy is often mandatory after partial splenectomy in patients with spherocytosis. In the work of Hafezi et al., this risk was 39% in patients not previously subjected to cholecystectomy, much higher than in patients who underwent total splenectomy. This suggests that the residual hemolytic splenic activity in the patients with partial splenectomy makes them more susceptible to the onset of gallbladder gallstones [39].

In many studies, a regrowth of the residual spleen was reported after the intervention of partial splenectomy. In the study by Rice et al., among 13 patients that underwent partial splenectomy, the volume of the spleen remained between 15 and 30% of the initial volume after two years, up to 40% after four years. In addition, in four patients where growth was greater than 75%, only two episodes of hemolysis occurred later, suggesting that this event is not related to a return of the disease [37]. Further studies on the risk of a second surgery should certainly be carried out, even if the advantages linked to the maintenance of the immune function of the spleen (even if transient) seem to be higher than the risk of an additional surgical procedure [44].

## 5. Conclusions

Partial open or laparoscopic splenectomy is a safe procedure associated with a low incidence of morbidity and it has become an important option in children with hematologic disorders that require the removal of the spleen.

The possibility of partially preserving splenic tissue seems to ensure a reduction of the main infectious risks related to splenectomy, and an adequate hematologic response, even if such evidence should be supported by more comprehensive future trials. However, although in the absence of reliable data, it is necessary to recommend vaccination and antibiotic prophylaxis also to those patients who undergo partial splenectomy.

## Figures and Tables

**Table 1:** Medical and surgical indications to splenectomy.

**MEDICAL INDICATIONS**
*Congenital hemolytic anemia* Hereditary spherocytosis Hereditary ellipsocitosis Pyruvate kinase deficiency Drepanocytosis
*Acquired immunological disorders* Autoimmune platelets Autoimmune hemolytic anemia
*Hypersplenism* Thalassemia Congestive splenomegaly Accumulation disorder (Gaucher disease, Niemann-Pick disease)
**SURGICAL INDICATIONS**
*Traumatic rupture of the spleen*
*Intrinsic splenic diseases* Cysts Hemangioma Lymphoma
*Surgery in the left hemiabdomen*

**Table 2:** Main information for the family and patients’ education.

The risk of infection increases throughout life.
It is important to inform your caregiver of the state of asplenia.
Perform a medical evaluation before traveling especially to malaria-endemic areas.
Always have an antibiotic on hand.
Perform vaccinations against the main capsulated germs (Pneumococcus, Meningococcus and Hemophilus Influenzae type B, and annual anti-flu vaccination)
Be very careful about animal bites or insect bites.
The patient should be informed about symptoms that may indicate the onset of OPSI (high fever, myalgia, headache, vomiting, abdominal pain, chills).
Suggest registering in a register of patients with diabetes (*if available*).
Carry an identification plate with the indication of the condition of splenectomized.

## References

[1] Di Buono G., Maienza E., Buscemi S., Gulotta L., Romano G., Agrusa A. (2020). Laparoscopic near-total splenectomy. Report of a case. Int. J. Surg. Case. Rep..

[2] Diesen D.L., Zimmerman S.A., Thornburg C.D., Ware R.E., Skinner M., Oldham K.T., Rice H.E. (2008). Partial splenectomy for children with congenital hemolytic anemia and massive splenomegaly. J. Pediatr. Surg..

[3] Bickenbach K.A., Gonen M., Labow D.M., Strong V., Heaney M.L., Zelenetz A.D., Coit D.G. (2013). Indications for and efficacy of splenectomy for haematological disorders. Br. J. Surg..

[4] Belli A.K., Dönmez C., Özcan Ö., Dere Ö., Dirgen Çaylak S., Dinç Elibol F., Yazkan C., Yılmaz N., Nazlı O. (2018). Adherence to vaccination recommendations after traumatic splenic injury. Ulus. Travma. Acil. Cerrahi. Derg..

[5] Williams L., Warwick R., Dyson M., Bannister L.H. (1989). Gray’s Anatomy.

[6] Tahir F., Ahmed J., Malik F. (2020). Post-splenectomy Sepsis: A Review of the Literature. Cureus.

[7] Lee G.M. (2020). Preventing infections in children and adults with asplenia. Hematol. Am. Soc. Hematol. Educ. Program.

[8] Chaturvedi S., Arnold D.M., McCrae K.R. (2018). Splenectomy for immune thrombocytopenia: Down but not out. Blood.

[9] Rab M.A.E., Meerveld-Eggink A., van Velzen-Blad H., van Loon D., Rijkers G.T., de Weerdt O. (2018). Persistent changes in circulating white blood cell populations after splenectomy. Int. J. Hematol..

[10] Leone G., Pizzigallo E. (2015). Bacterial Infections Following Splenectomy for Malignant and Nonmalignant Hematologic Diseases. Mediterr. J. Hematol. Infect. Dis..

[11] Chong J., Jones P., Spelman D., Leder K., Cheng A.C. (2017). Overwhelming post-splenectomy sepsis in patients with asplenia and hyposplenia: A retrospective cohort study. Epidemiol. Infect..

[12] Dionne B., Dehority W., Brett M., Howdieshell T.R. (2017). The Asplenic Patient: Post-Insult Immunocompetence, Infection, and Vaccination. Surg. Infect..

[13] Salvadori M.I., Price V.E., Canadian Paediatric Society, Infectious Diseases and immunization Committee (2014). Preventing and treating infections in children with asplenia or hyposplenia. Paediatr. Child Health.

[14] Madenci A.L., Armstrong L.B., Kwon N.K., Jiang W., Wolf L.L., Koehlmoos T.P., Ricca R.L., Weldon C.B., Haider A.H., Weil B.R. (2019). Incidence and risk factors for sepsis after childhood splenectomy. J. Pediatr. Surg..

[15] Lewis S.M., Williams A., Eisenbarth S.C. (2019). Structure and function of the immune system in the spleen. Sci. Immunol..

[16] de Porto A.P., Lammers A.J., Bennink R.J., ten Berge I.J., Speelman P., Hoekstra J.B. (2010). Assessment of splenic function. Eur. J. Clin. Microbiol. Infect. Dis..

[17] Buzelé R., Barbier L., Sauvanet A., Fantin B. (2016). Medical complications following splenectomy. J. Visc. Surg..

[18] Kruetzmann S., Rosado M.M., Weber H., Germing U., Tournilhac O., Peter H.H., Berner R., Peters A., Boehm T., Plebani A. (2003). Human immunoglobulin M memory B cells controlling Streptococcus pneumoniae infections are generated in the spleen. J. Exp. Med..

[19] Davies J.M., Lewis M.P., Wimperis J., Rafi I., Ladhani S., Bolton-Maggs P.H. (2011). British Committee for Standards in Haematology. Review of guidelines for the prevention and treatment of infection in patients with an absent or dysfunctional spleen: Prepared on behalf of the British Committee for Standards in Haematology by a working party of the Haemato-Oncology task force. Br. J. Haematol..

[20] El-Alfy M.S., El-Sayed M.H. (2004). Overwhelming postsplenectomy infection: Is quality of patient knowledge enough for prevention?. Hematol. J..

[21] Casciani F., Trudeau M.T., Vollmer C.M. (2020). Perioperative Immunization for Splenectomy and the Surgeon’s Responsibility: A Review. JAMA Surg..

[22] Ruggiero A., Battista A., Coccia P., Attinà G., Riccardi R. (2011). How to manage vaccinations in children with cancer. Pediatr. Blood. Cancer.

[23] Luu S., Spelman D., Woolley I.J. (2019). Post-splenectomy sepsis: Preventative strategies, challenges, and solutions. Infect. Drug. Resist..

[24] Williamson E.J., Walker A.J., Bhaskaran K., Bacon S., Bates C., Morton C.E., Curtis H.J., Mehrkar A., Evans D., Inglesby P. (2020). Factors associated with COVID-19-related death using Open SAFELY. Nature.

[25] Ruggiero A., Attinà G., Chiaretti A. (2020). Additional hypotheses about why COVID-19 is milder in children than adults. Acta Paediatr..

[26] Cullingford G.L., Watkins D.N., Watts A.D., Mallon D.F. (1991). Severe late postsplenectomy infection. Br. J. Surg..

[27] Rothman J.A., Stevens J.L., Gray F.L., Kalfa T.A. (2020). How I approach hereditary hemolytic anemia and splenectomy. Pediatr. Blood Cancer.

[28] Boam T., Sellars P., Isherwood J., Hollobone C., Pollard C., Lloyd D.M., Dennison A.R., Garcea G. (2017). Adherence to vaccination guidelines post splenectomy: A five year follow up study. J. Infect. Public Health.

[29] Liu G., Fan Y. (2019). Feasibility and Safety of Laparoscopic Partial Splenectomy: A Systematic Review. World J. Surg..

[30] Zhang L., Yan P., Yang K., Wu S., Bai Y., Zhu X., Chen X., Li L., Cao Y., Zhang M. (2021). Association between splenectomy and chronic thromboembolic pulmonary hypertension: A systematic review and meta-analysis. BMJ Open.

[31] Stoehr G.A., Stauffer U.G., Eber S.W. (2005). Near-total splenectomy: A new technique for the management of hereditary spherocytosis. Ann. Surg..

[32] Catalano M., Rizzo D., Coccia P., Maurizi P., Nanni L., Ruggiero A. (2018). Can partial splenectomy preserve humoral immunity in pediatric patients? Risks and benefits of partialsplenectomy. Signa. Vitae..

[33] Koren A., Haasz R., Tiatler A., Katzuni E. (1984). Serum immunoglobulin levels in children after splenectomy. A prospective study. Am. J. Dis. Child..

[34] Sheikha A.K., Salih Z.T., Kasnazan K.H., Khoshnaw M.K., Al-Maliki T., Al-Azraqi T.A., Zafer M.H. (2007). Prevention of overwhelming postsplenectomy infection in thalassemia patients by partial rather than total splenectomy. Can. J. Surg..

[35] Buesing K.L., Tracy E.T., Kiernan C., Pastor A.C., Cassidy L.D., Scott J.P., Ware R.E., Davidoff A.M., Rescorla F.J., Langer J.C. (2011). Partial splenectomy for hereditary spherocytosis: A multi-institutional review. J. Pediatr. Surg..

[36] Guizzetti L. (2016). Total versus partial splenectomy in pediatric hereditary spherocytosis: A systematic review and meta-analysis. Pediatr. Blood Cancer.

[37] Rice H.E., Oldham K.T., Hillery C.A., Skinner M.A., O’Hara S.M., Ware R.E. (2003). Clinical and hematologic benefits of partial splenectomy for congenital hemolytic anemias in children. Ann. Surg..

[38] Englum B.R., Rothman J., Leonard S., Reiter A., Thornburg C., Brindle M., Wright N., Heeney M.M., Smithers C.J., Brown R.L. (2016). Splenectomy in Congenital Hemolytic Anemia Consortium. Hematologic outcomes after total splenectomy and partial splenectomy for congenital hemolytic anemia. J. Pediatr. Surg..

[39] Hafezi N., Carpenter K.L., Colgate C.L., Gray B.W., Rescorla F.J. (2021). Partial splenectomy in children: Long-term preoperative outcomes. J. Pediatr. Surg.

[40] Timeus F., Crescenzio N., Longoni D., Doria A., Foglia L., Pagliano S., Vallero S., Decimi V., Svahn J., Palumbo G. (2014). Paroxysmal nocturnal hemoglobinuria clones in children with acquired aplastic anemia: A multicentre study. PLoS ONE.

[41] Rinninella E., Ruggiero A., Maurizi P., Triarico S., Cintoni M., Mele M.C. (2017). Clinical tools to assess nutritional risk and malnutrition in hospitalized children and adolescents. Eur. Rev. Med. Pharmacol. Sci..

[42] Romboli A., Annicchiarico A., Morini A., Castro Ruiz C., Pagliai L., Montali F., Costi R. (2021). Laparoscopic Partial Splenectomy: A Critical Appraisal of an Emerging Technique. A Review of the First 457 Published Cases. J. Laparoendosc. Adv. Surg. Tech. A.

[43] Morinis J., Dutta S., Blanchette V., Butchart S., Langer J.C. (2008). Laparoscopic partial vs total splenectomy in children with hereditary spherocytosis. Pediatr. Surg..

[44] Riera M., Buczacki S., Khan Z.A. (2009). Splenic regeneration following splenectomy and impact on sepsis: A clinical review. J. R. Soc. Med..

